# Human antibody recognition of antigenic site IV on Pneumovirus fusion proteins

**DOI:** 10.1371/journal.ppat.1006837

**Published:** 2018-02-22

**Authors:** Jarrod J. Mousa, Elad Binshtein, Stacey Human, Rachel H. Fong, Gabriela Alvarado, Benjamin J. Doranz, Martin L. Moore, Melanie D. Ohi, James E. Crowe

**Affiliations:** 1 Vanderbilt Vaccine Center, Vanderbilt University Medical Center, Nashville, Tennessee, United States of America; 2 Department of Cell and Developmental Biology, Vanderbilt University Medical Center, Nashville, Tennessee, United States of America; 3 Department of Pediatrics, Emory University School of Medicine, Atlanta, Georgia, United States of America; 4 Children’s Healthcare of Atlanta, Atlanta, Georgia, United States of America; 5 Integral Molecular Inc., Philadelphia, Pennsylvania, United States of America; 6 Department of Pathology, Microbiology and Immunology, Vanderbilt University, Nashville, Tennessee, United States of America; 7 Department of Pediatrics, Vanderbilt University School of Medicine, Nashville, Tennessee, United States of America; The Peter Doherty Institute and Melbourne University, AUSTRALIA

## Abstract

Respiratory syncytial virus (RSV) is a major human pathogen that infects the majority of children by two years of age. The RSV fusion (F) protein is a primary target of human antibodies, and it has several antigenic regions capable of inducing neutralizing antibodies. Antigenic site IV is preserved in both the pre-fusion and post-fusion conformations of RSV F. Antibodies to antigenic site IV have been described that bind and neutralize both RSV and human metapneumovirus (hMPV). To explore the diversity of binding modes at antigenic site IV, we generated a panel of four new human monoclonal antibodies (mAbs) and competition-binding suggested the mAbs bind at antigenic site IV. Mutagenesis experiments revealed that binding and neutralization of two mAbs (3M3 and 6F18) depended on arginine (R) residue R429. We discovered two R429-independent mAbs (17E10 and 2N6) at this site that neutralized an RSV R429A mutant strain, and one of these mAbs (17E10) neutralized both RSV and hMPV. To determine the mechanism of cross-reactivity, we performed competition-binding, recombinant protein mutagenesis, peptide binding, and electron microscopy experiments. It was determined that the human cross-reactive mAb 17E10 binds to RSV F with a binding pose similar to 101F, which may be indicative of cross-reactivity with hMPV F. The data presented provide new concepts in RSV immune recognition and vaccine design, as we describe the novel idea that binding pose may influence mAb cross-reactivity between RSV and hMPV. Characterization of the site IV epitope bound by human antibodies may inform the design of a pan-Pneumovirus vaccine.

## Author summary

Respiratory syncytial virus (RSV) and human metapneumovirus (hMPV) are major causes of viral bronchiolitis and pneumonia in infants and children. A licensed vaccine is not currently available for either virus, therefore, the characterization of the human antibody response to these viruses is of the utmost importance. In this study, we identified four new human monoclonal antibodies that target antigenic site IV of the RSV F protein. Using a combination of alanine mutagenesis, protein binding experiments, and electron microscopy, we defined several modes of antibody recognition at antigenic site IV, including a mAb with a unique binding angle that neutralizes RSV and hMPV. The data presented provide new concepts that will aid in the development of vaccines for RSV and hMPV.

## Introduction

Viral bronchiolitis consistently remains a burden among young children. Respiratory syncytial virus (RSV) is chief among these infections as the leading cause of viral bronchiolitis and viral pneumonia in infants and children [1,2]. The related pneumovirus, human metapneumovirus (hMPV), contributes to this burden, with infection resulting in 5–10% of hospitalizations due to lower respiratory tract infections [3–5]. hMPV was first identified in 2001, yet it is thought to have infected the human population for at least fifty years [6]. As major human pathogens, RSV and hMPV are among the few infectious viruses with global impact for which there is no licensed vaccine. Palivizumab [7] (Synagis) has become the standard of care for prophylactic treatment against RSV, yet the availability and effectiveness of palivizumab for preventing disease is limited. Furthermore, a second generation antibody candidate, motavizumab, has been developed with higher affinity and more potent neutralization, but was not approved for prophylactic use [8]. Although palivizumab is accessible for prophylactic RSV treatment, such a treatment is not available for hMPV infection.

Recent attempts to develop an RSV vaccine have focused on the highly conserved fusion (F) protein, a type I F protein that has both pre- and post-fusion conformations [9,10]. Pneumovirus F proteins are synthesized as inactive precursors (F_0_) that are cleaved during cellular processing into two disulfide-linked domains (F_1_ and F_2_). Upon activation, the F proteins cause fusion of viral and cell membranes. The RSV F pre-fusion conformation is meta-stable, easily transitioning to the post-fusion conformation upon viral attachment to host cells. Recombinant RSV F protein readily converts to the post-fusion conformation [11], and formalin inactivation of RSV was shown to result in the pre- to post-fusion rearrangement of the fusion protein [12]. Stabilization of the RSV F protein in the pre-fusion conformation has proven successful, resulting in Ds-Cav1 (disulfide-linked, cavity-filled) and SC-TM (single-chain-triple mutant) variants with enhanced stability in recombinant expression [13,14]. Both pre-fusion variants have been characterized structurally, as has post-fusion RSV F [11].

Several major neutralizing epitopes exist on RSV F, based on functional and structural data (S1 Fig). Antigenic site II [15] recognized by the humanized murine mAbs palivizumab and motavizumab, and antigenic site IV [16] recognized by murine mAb 101F, are preserved in both the pre-fusion and post-fusion RSV F conformations. Pre-fusion-specific mAbs have been characterized that defined new antigenic sites including mAbs D25 (site Ø) [17], MPE8 (site III) [18,19], and the recently discovered mAb hRSV90 (site VIII) [20]. A quaternary epitope has been described by recognition via human mAb AM14 [21]. Several antibodies targeting the hMPV F protein have been isolated using murine hybridoma or phage display techniques, and the known hMPV F antigenic sites appear structurally similar to those on RSV F [22–24]. A monomeric hMPV F protein has been characterized structurally in complex with a neutralizing antibody DS7 [25], and in the post-fusion conformation [26].

RSV and hMPV F share ~36% sequence homology, and human antibodies that cross-neutralize RSV and hMPV have been described [18,19,26–28]. The human mAb MPE8 was found to neutralize four different pneumoviruses, including RSV, hMPV, bovine RSV, and pneumonia virus of mice [18], and a similar mAb 25P13 has been described [19]. The human mAb 54G10 recognizes the site IV region of the RSV F protein, cross-reacts with hMPV F protein, and protects against viral infection *in vivo* [27]. Of the antigenic sites described thus far, site IV consists partially of a predominantly linear region demonstrated in the co-crystallization of the mouse-derived mAb 101F in complex with a 15-mer peptide [16]. MAb 101F also was shown to cross-react with both RSV and hMPV [26]. We recently reported several human antibodies generated from human B cells that primarily target antigenic sites II and VIII [15,20]. However, structural and functional data regarding human mAb binding to antigenic site IV is lacking. Recent work described the generation and characterization of over 300 human mAbs generated via B cell sorting, and those mAbs targeting antigenic site IV were recognized as a substantial percentage (~15–40%) in the human repertoire [28]. Previous reports have described binding of the murine-derived mAb 101F [16,26,29], as well as several binding sites (IV, V, VI) near the site IV epitope [9]. To further define the molecular basis for human antibody site IV-mediated binding, as well as the cross-reactivity between RSV and hMPV at antigenic site IV, we generated additional human antibodies against post-fusion RSV F. Herein, we describe four of these mAbs that were specific to antigenic site IV, including the identification of several binding modes at antigenic site IV.

## Results

### Generation and epitope specificity of RSV F-specific human monoclonal antibodies

To understand binding modes at antigenic site IV by human antibodies, we generated new RSV F-specific human mAbs using human hybridoma technology [30,31]: designated mAbs 2N6, 3M3, 6F18, and 17E10. These mAbs were generated similar to those previously described to the RSV F protein [15,19,20]. Half maximal effective concentration (EC_50_) for each mAb was measured by enzyme-linked immunosorbent assay (ELISA) (Table 1, S2 Fig). Each mAb had comparable binding to F proteins representing viruses across the RSV antigenic subgroups, using RSV A strain A2 and RSV B strain 18537, as well as pre-fusion [single chain-triple mutant (SC-TM) and disulfide-cavity filling (DsCav1)] and post-fusion RSV F proteins. These findings suggested the epitope for the generated mAbs is present in both pre-fusion and post-fusion conformations. The mAbs were tested for neutralization of RSV A2 and RSV Long viruses (subgroup A), and RSV 18537 B and RSV WV/401R viruses (subgroup B) (Table 1, S3 Fig). Each mAb neutralized all strains with one especially potent mAb designated 3M3 having a half maximal inhibitory concentration (IC_50_) of 3 ng/mL for the RSV Long strain. The mAb sequences were analyzed by IMGT [32], and each mAb is predicted to utilize a unique V gene sequence compared to each other (Table 2). Furthermore, the heavy chain CDR3 length varies between 14–17 amino acids, except for mAb 17E10, which contains just 8 amino acids.

**Table 1:** Binding and neutralization values for isolated RSV F-specific mAbs.

Monoclonal antibody	IgG subclass	Light chain	Binding EC_50_ (ng/mL)			Neutralization IC_50_ (ng/mL)			
			RSV A2	RSV A2 SC-TM	RSV 18537 B DSCAV1	RSV A2	RSV Long	RSV 18537 B	RSV WV/401R
**2N6**	1	λ	14	12	18	790	140	1,900	1,400
**3M3**	1	λ	20	13	20	14	3	16	29
**6F18**	1	κ	14	11	17	260	96	520	340
**17E10**	1	λ	11	13	15	2,200	334	1,000	15,00
**Control mAbs**									
**D25**	1	κ	>	16	>	20	6.1	1,600	33
**motavizumab**	1	κ	96	69	66	60	7.5	25	40
**101F**	1	κ	11	14	21	1,200	150	540	1,400
**54G10**	1	κ	19	130	44	>	>	>	>

EC_50_ values correspond to the concentration at which half-maximum signal was obtained in ELISA, based on optical density at 405 nm. Neutralization values were determined using a plaque-reduction assay, where the IC_50_ corresponds to the mAb concentration at which 50% plaque reduction was observed. > indicates no plaque reduction in neutralization assays or no signal detected below 25 μg/mL in ELISA binding assays. DS-Cav1 and SC-TM represent stabilized pre-fusion RSV F.

**Table 2:** Antibody sequence analysis for the V gene usage and CDR3 sequence.

mAb	Donor	Heavy chain			Light chain		
		V gene % identity	CDR3 AA length	CDR3 AA sequence	V gene % identity	CDR3 AA length	CDR3 AA sequence
**2N6**	1	IGHV4-34*02 78%	8.7.14	CAAPRRVDGYNLLDSW	IGLV1-40*01 94%	9.3.12	CQSFDNSRTAFYIF
**3M3**	2	IGHV4-34*08 91%	8.7.16	CSRGVADRISSSWHYDLW	IGLV3-21*02 87%	6.3.11	CQVWDMENDHPVF
**6F18**	1	IGHV6-1*01 91%	10.9.17	CARSQDDSSGYHEDFFDFW	IGKV4-1*01 96%	12.3.9	CQQYYRMPYTF
**17E10**	2	IGHV3-15*01 87%	8.10.8	CSVLYSLQHW	IGLV7-46*01 90%	9.3.9	CLLSYNNMLVF

Analysis was carried out using IMGT/VQUEST [32]. The predicted V gene usage is displayed for both heavy chain and light chain, along with the CDR3 sequence and length based on the amino acid (AA) sequence.

**Fig 1:**
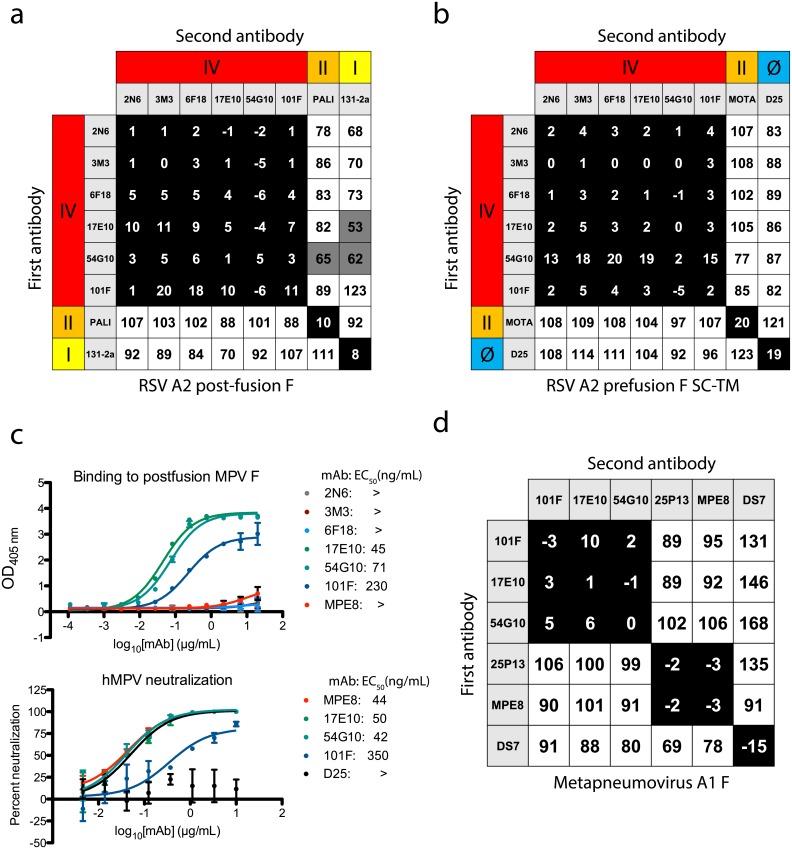
Epitope binning and hMPV F cross-reactivity. (A) Epitope binning with the newly generated mAbs on post-fusion RSV A2 F protein revealed the mAbs competed for binding to antigenic site IV with the previously described mAbs 101F and 54G10. (B) Epitope binning on pre-fusion RSV A2 F SC-TM suggested binding at antigenic site IV, as competition was not observed with site II mAb motavizumab nor site Ø mAb D25. (C) Binding and neutralization curves indicate mAb 17E10 cross-reacts with hMPV F. The top graph displays ELISA binding data with post-fusion hMPV A1 F protein. Only cross-reactive mAbs 17E10, 54G10, 101F, and MPE8 show binding to the protein, while the site IV mAbs 2N6, 3M3, and 6F18 show no binding. Each data point is the average of three independent experiments, each with four technical replicates. Error bars represent the standard deviation. The bottom graph show neutralization for the cross-reactive mAbs, with D25 used as a negative control. MAbs 17E10, MPE8, and 101F neutralize hMPV F while the D25 control shows no reduction in virus. Data points are the average of three technical replicates, and error bars indicate the standard deviation. (D) Epitope binning using post-fusion hMPV F. MAbs 101F, 17E10, and 54G10 display a similar competition binding pattern to that observed with RSV F protein. The mAbs compete for a site unique from site III mAbs 25P13 and MPE8, and DS7. For epitope binning, data indicate the percent binding of the second antibody in the presence of the first antibody, compared with the second antibody alone. Cells filled in black indicate full competition, in which ≤33% of the uncompeted signal was observed, intermediate competition (gray) if signal was between 33% and 66%, and noncompeting (white) if signal was ≥66%. Antigenic sites are highlighted at the top and side based on competition-binding with the control mAbs D25 (site Ø), 131-2a (site I), palivizumab (PALI) or motavizumab (MOTA) (site II), or 101F (site IV).

The generated mAbs were tested for epitope specificity using a biolayer interferometry-based competition-binding assay [15,20]. For this purpose, post-fusion RSV F or the pre-fusion-stabilized SC-TM variant [13] were loaded onto sensor tips, and mAbs were competed for binding to each protein. In measuring competition on the post-fusion RSV F protein, the isolated mAbs share a unique antigenic site that did not overlap with either site II (recognized by palivizumab) or site I (recognized by murine mAb 131-2a) (Fig 1A). Instead, the mAbs competed with each other, the previously described mouse-human chimeric mAb 101F [29], and the human mAb 54G10 [27], both of which target antigenic site IV. When loading sensors with pre-fusion RSV F A2 SC-TM protein, the pre-fusion-specific mAb D25 [17] was used to confirm the presence of the pre-fusion conformation, and to identify site Ø (Fig 1B). As expected, the site Ø mAb D25 did not compete with the site IV mAbs nor motavizumab at site II. Competition-binding patterns were consistent in assays using F protein in either pre-fusion or post-fusion conformation, further suggesting the mAbs target antigenic site IV, as this epitope is retained in both conformations of the F protein.

### Multiple binding modes at antigenic site IV

The previously described site IV mAb 101F was reported to neutralize both RSV and hMPV [26]. Similarly, mAb 54G10 neutralizes hMPV, as well as RSV at high concentrations, while also reducing hMPV and RSV titers *in vivo* [27]. As the newly generated mAbs competed for binding to F with both of these known RSV/hMPV cross-reactive mAbs, we tested whether the newly isolated mAbs were cross-reactive to hMPV F by first testing binding in ELISA to the hMPV F protein. The mAb designated 17E10 bound to hMPV F, while the remaining three mAbs did not show detectable binding when tested at concentrations up to 20 μg/mL (Fig 1C). The cross-reactive mAb 17E10 neutralized hMPV infection in a neutralization assay at a level comparable to that of the site IV mAb 54G10, and the site III mAb MPE8 (Fig 1C). To confirm binding near site IV on hMPV F, competition-binding studies were performed using recombinant post-fusion hMPV F protein. Indeed, mAb 17E10 competed with mAbs 101F and 54G10, but its pattern of competition differed from that of the site III mAbs MPE8 and 25P13 [19], and the hMPV F-specific mAb DS7 Fab [25] (Fig 1D). These data suggest that there are several distinct epitopes within antigenic site IV, and particular interactions or binding poses can induce cross-reactivity with hMPV F.

**Fig 2:**
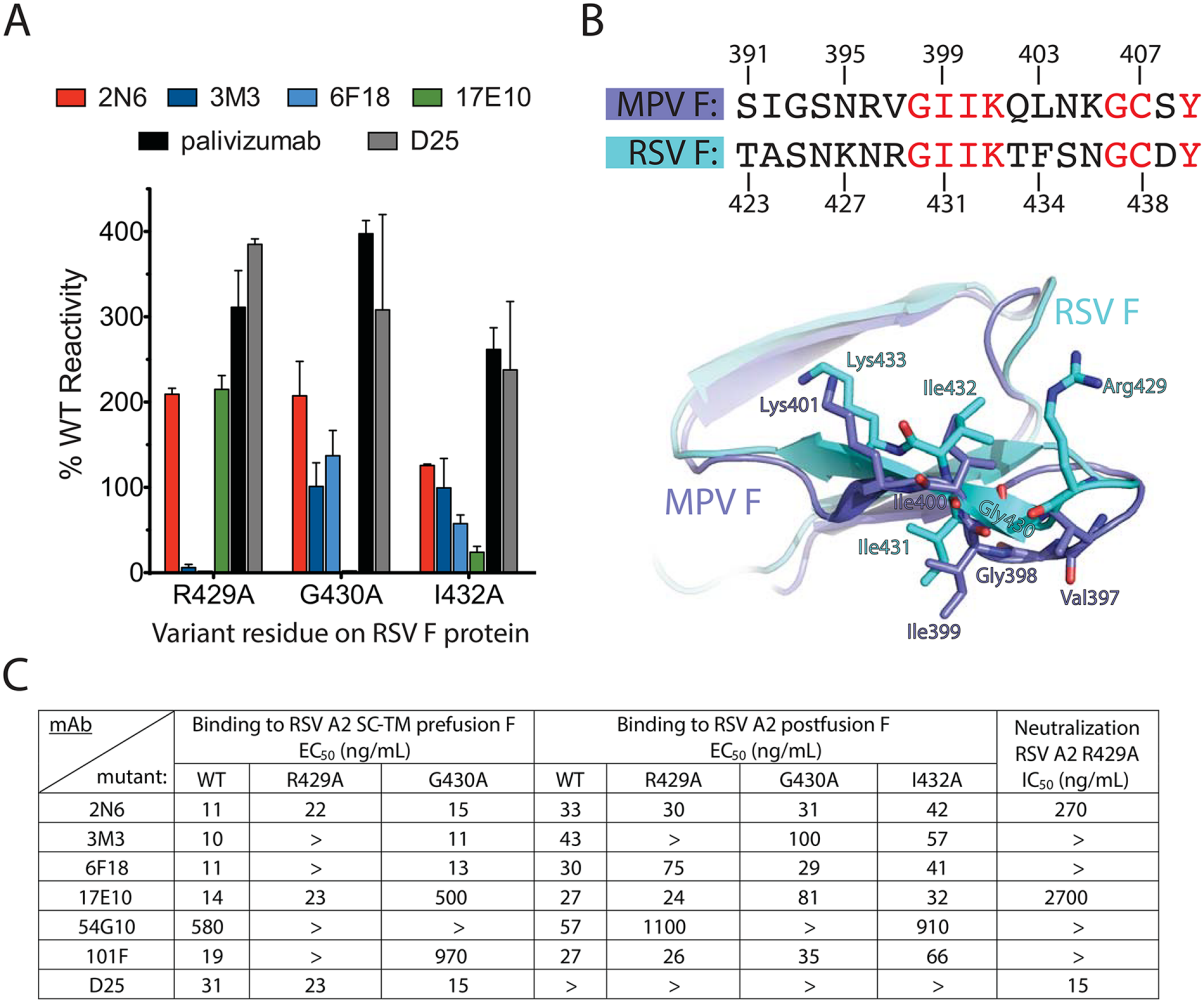
Characterization of antigenic site IV mutations. (A) Alanine-scanning mutagenesis binding values for the generated site IV mAbs, compared with palivizumab and mAb D25 controls. The mAb reactivity for each RSV F variant was calculated relative to that of wild-type RSV F. Error bars indicate the measurement range of two independent experiments. (B) Overlay of RSV F and hMPV F sequences and crystal structures (PDB IDs: 3RRR, 5L1X, overlaid at chain H for each structure) at antigenic site IV, with RSV F residues from the alanine-scanning mutagenesis shown. Conserved residues between RSV F and hMPV F are displayed in red font. In the crystal structure overlay, RSV F residues are shown in cyan and hMPV F residues are shown in blue. (C) ELISA EC_50_ values for recombinant post-fusion or pre-fusion (SC-TM) mutant proteins for the site IV mAbs or controls. Neutralization IC_50_ values also are displayed for the RSV strain A2 F variant R429A.

To determine residues important for the generated mAbs, we used an alanine-scanning mutagenesis F protein library expressed in HEK-293T cells coupled with flow cytometric detection of mAb binding or loss of binding. Each RSV F construct was transfected and allowed to express for 16 h before fixing cells with 4% (vol/vol) paraformaldehyde. The fixed cells were incubated with each mAb, followed by incubation with a fluorescent secondary antibody, and cellular fluorescence was detected by flow cytometry. From the library, no mutant disrupted binding of mAb 2N6 significantly, suggesting no single residue alone is critical for binding. The R429 residue that has been previously identified as the principal contact residue for mAbs recognizing antigenic site IV was critical for binding of mAbs 3M3 and 6F18, yet this residue did not affect binding of mAbs 17E10 or 2N6 (Fig 2A, S4 Fig). Instead, G430A and I432A mutants resulted in loss of mAb 17E10 binding to RSV F. All three mutations were shown previously to reduce binding of mAb 101F to cell surface-expressed proteins [33]. Sequence alignment of the RSV and hMPV F proteins revealed a conserved amino acid motif of GIIK at antigenic site IV (Fig 2B), while the corresponding R429 residue in hMPV F is replaced by a valine in the hMPV protein. Thus, we hypothesized that amino acids within the conserved GIIK sequence were important for the cross-reactivity of mAb 17E10.

We further investigated this hypothesis using recombinantly expressed post-fusion RSV F mutant proteins postF-R429A, postF-G430A, or postF-I432A. Binding of each of the new site IV mAbs was tested by ELISA and, as expected, 3M3 failed to bind the postF-R429A mutant (Figs 2C and S5). MAb 6F18 retained binding to the postF-R429A protein, different from the results where binding was lost to cell-surface expressed mutant protein (Fig 2A and 2C; S4 and S5 Figs). Another difference observed was the binding of 17E10 to the postF-G430A and postF-I432A mutants, which did not abrogate binding. These differences can be explained by the presence of the pre-fusion conformation in the cell surface-expressed system, while the recombinant protein is in the post-fusion conformation.

To account for these differences, we generated site IV residue mutants in the pre-fusion RSV A2 F SC-TM construct, which were confirmed via binding of mAb D25. In this case, binding matched data from the cell surface-expressed system, where binding for 6F18 was now lost at R429A, and 17E10 had reduced binding to the G430A mutant. Similarly, the R429A mutation abolished 101F binding only to F in the pre-fusion conformation but not to post-fusion F. The R429 residue was shown to bind deep into the antibody heavy/light chain interface of 101F in the reported crystal structure [16], and to be important for binding of 101F to a site IV-derived peptide [29]. The pre-fusion I432A mutant could not be tested as protein could not be obtained despite multiple expression attempts. The cross-reactive mAb 54G10 had reduced binding to the postF-R429A and postF-G430A mutants, and the preF-G430A mutant. Based on these data, it appears the mAbs 54G10, 6F18, 17E10, and 101F have additional contacts in post-fusion RSV F, which are absent in pre-fusion RSV F.

To clarify the differential binding between cell-surface expressed F, pre-fusion F, and post-fusion F, we generated a recombinant RSV R429A escape mutant virus and tested neutralization for each of the mAbs (Fig 2C; S6 Fig). As expected, mAb 6F18, 101F, and 3M3 did not neutralize the virus, while neutralizing activity was retained for mAbs 17E10 and 2N6, consistent with the pre-fusion F R429A ELISA binding data. We could not rescue mutants for G430A or I432A.

**Fig 3:**
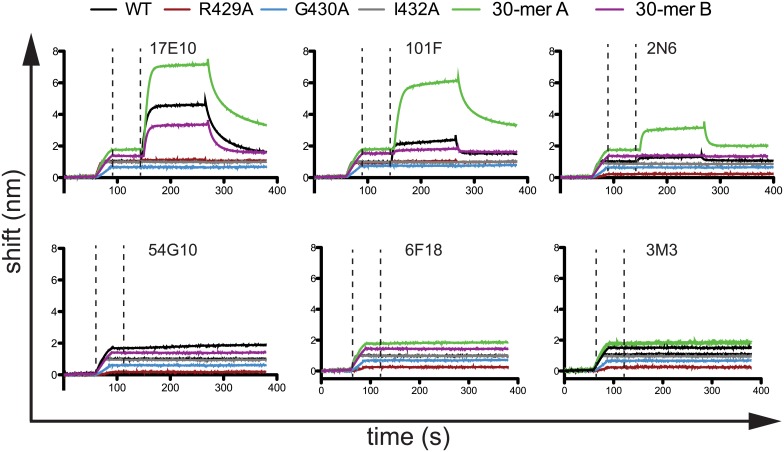
Binding curves determined by biolayer interferometry for mAbs targeting antigenic site IV. Streptavidin biosensors were incubated with biotinylated peptides, and then mAbs were tested for binding in real-time. The first dashed line indicates the end of the peptide loading step, and the second dashed line indicates the beginning of the mAb binding step. After binding, mAbs were allowed to dissociate in real-time. The data are representative chromatograms from one experiment. Wild-type (WT) and mutant peptides consist of RSV F residues 422–436. The 30-mer A peptide includes RSV F residues 407–436, and the 30-mer B peptide includes RSV F residues 422–451.

As antigenic site IV consists at least partially of a linear epitope, peptides have been used previously to characterize mAb binding [16]. Using a set of synthesized biotinylated 15-mer peptides consisting of residues 422–436, we tested binding of 2N6, 3M3, 6F18, 17E10, 101F, and 54G10, using streptavidin-coupled biosensors and streptavidin-coated ELISA plates. Only mAbs 17E10 and 101F bound the wild-type 15-mer peptide by biosensor or ELISA (Fig 3; S7 Fig). Mutant peptides containing R429A, G430A, or I432A mutations abolished binding of both 17E10 and 101F. Although the R429 residue was found to be nonessential for mAb 17E10 binding, it is likely the mAb makes other contacts outside of the 15-mer region that are important for binding in full-length RSV F. Since the other RSV F-specific mAbs failed to bind to the peptides, peptides extending 15 residues in either N-terminal or C-terminal direction consisting of residues 407–436 (30-mer A) and 422–451 (30-mer B) were used to identify binding residues flanking the 15-mer region. For the 30-mer A peptide, binding was significantly enhanced for mAbs 17E10 and 101F, and binding was now observed for mAb 2N6 (Fig 3). The 30-mer B peptide did not have this effect, suggesting additional contact residues for 17E10, 101F, and 2N6 extend in the N-terminal direction. As the 30-mer B did not affect binding, it is unlikely the increased binding to the 30-mer A peptide was due to increased peptide length. It is worth noting that only mAbs 17E10 and 101F bound to the 15-mer peptide, as this suggests that cross-reactive mAbs target this conserved area. However, it is clear that peptides to not properly recapitulate binding to recombinant proteins for antigenic site IV.

### Electron microscopy determines the structural basis for cross-reactivity

**Fig 4:**
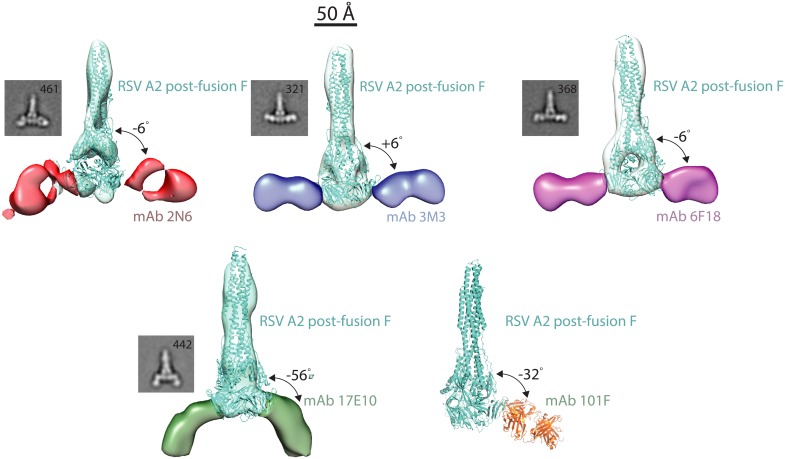
Multiple binding modes at antigenic site IV. Each site IV mAb was complexed with RSV A2 post-fusion F, and negative-strain electron microscopy images were generated using random-conical tilt analysis. MAbs 3M3 and 6F18 share a similar binding mode, while mAb 2N6 binds antigenic site IV at an angle allowing bypass of the Arg429 residue. MAb 17E10 binds to RSV F at an angle 42° from the plane of the other site IV mAbs. This unique binding pose likely mediates cross-reactivity with hMPV. 3-D reconstructions are displayed for each mAb-RSV F complex, and 2D class averages are displayed below the reconstructions. The X-ray crystal structure of the 101F-peptide complex (PDB ID: 3O41) was aligned to the antigenic site IV region on the post-fusion RSV F crystal structure (3RRR).

To further probe the structural basis for the different binding motifs and the mAb cross-reactivity with hMPV F, we generated complexes of the new mAbs 2N6, 3M3, 6F18, and 17E10 with post-fusion RSV F protein and generated 3-D reconstructions by negative-stain electron microscopy (Fig 4). The EM reconstructions include an elongated post-fusion RSV F protein with two fragment-antigen binding domains bound to the viral protein. Binding poses for mAbs 3M3 and 6F18, both of which rely on R429 for binding, were very similar. The two mAbs formed T-shaped complexes with the wide-axis of the Fab molecule perpendicular to the long-axis of post-fusion RSV F. In the case of mAb 2N6, an alternative binding pose was observed, as the Fab is rotated approximately 90° from the orientation of Fabs 3M3 and 6F18. These data help explain the retention of binding of mAb 2N6 to all mutants tested, as the unique binding pose allows contact residues outside of the canonical antigenic site IV. Finally, the complex of Fab 17E10 with RSV F was quite different from that of the others, with a binding angle 42° shifted from the other mAbs. This binding angle of 17E10 may be indicative of cross-reactivity between RSV and MPV, while also allowing mAb 17E10 to bind and neutralize RSV strains, including the R429A mutant. A similar binding angle shifted from the 90° line was observed for the murine-derived mAb 101F to RSV F [26] (Fig 4), and mAb cross-reactivity with hMPV F may be partially defined by this binding pose.

## Discussion

Here we define the complexity of antigenic site IV on the RSV F protein by identifying a panel of neutralizing human mAbs, each with diverse binding modes. The idea presented that mAbs targeting the same antigenic site can have highly diverse binding modes likely applies to all antibody-antigen interactions. Antigenic site IV is preserved in both pre-fusion and post-fusion RSV F. However, amino acids surrounding antigenic site IV change dramatically between the two conformations. Therefore, binding of mAbs at this site can be quite diverse depending on whether a mAb is tested for binding to pre-fusion or post-fusion F. A summary of the diverse binding characteristics of each mAb is shown in S1 Table. Previous studies suggested antigenic site IV site adopts a mostly linear conformation, and the corresponding antibodies appeared to form a single class of RSV-specific neutralizing antibodies. We find, in contrast, that human antibodies to this site recognize epitopes in diverse manners, and the potency and breadth of the neutralizing activity of those antibodies is associated with the fine details of the epitope, the binding angle, and other features of the antibody-antigen interaction. To understand the binding modes at antigenic site IV, we generated a panel of new antibodies and present four mAbs that target antigenic site IV mAbs, each with unique binding requirements. Of those new clones, mAb 3M3 is quite potent, and mAb 17E10 is cross-reactive with hMPV F. We cannot define the original ontogeny of mAb 17E10 as the donors were adults likely who have been infected multiple times with RSV and hMPV, thus the antibody could have been generated originally to RSV F or hMPV F, and then further mutated during subsequent infections. In general, mAbs are generated and optimized based on binding, as the germinal center reaction does not select for neutralizing capacity during affinity maturation. MAb 17E10 does neutralize hMPV much better than RSV, yet this does not prove that it was generated in response to hMPV. Rather, it is most clear that 17E10 binds to hMPV F in an orientation that facilitates more efficient inhibition of the pre- to post-fusion transition than for RSV F. This finding is similar to the previously discovered mAb 54G10, for which the extent of neutralization of hMPV and RSV were quite different. Reported IC_50_ values for mAb 54G10 were 60 ng/mL for hMPV B2 and 14,200 ng/mL for RSV A2 [27], similar values to those we observed in this report. The binding pose of 17E10, while facilitating cross-reactivity, appears to be less efficient at inhibiting the pre- to post-fusion transition for RSV F.

The amino acid R429 in RSV F has been the defining contact residue of site IV based on studies performed on the binding properties of antibodies isolated from previous phage display library experiments [34] and the murine/humanized-murine mAb 101F [29]. While the two mAbs 3M3 and 6F18 required R429 for binding, mAbs 17E10 and 2N6 bound to cell surface-expressed or recombinantly expressed protein independently of this residue. We also found site IV based cross-neutralization of RSV and hMPV is associated with recognition of the G430 and I432 residues, which are shared in RSV and hMPV F proteins, unlike R429, which is present only in RSV F. Furthermore, the binding poses of mAbs 17E10 and 2N6 show unique features allowing for the bypass of R429. No single amino acid was determined critical for binding of mAb 2N6, suggesting a mode of binding unique from mAbs 3M3, 6F18, 17E10, 101F, or 54G10. Further investigation of this binding mode is warranted through by X-ray crystallography.

MAb 17E10 binds in a pose much different than the other mAbs, which could determine cross-reactivity with hMPV. This pose is similar to that of the cross-reactive murine-derived mAb 101F, suggesting the tilted binding pose relative to antigenic site IV is indicative of cross-reactivity with hMPV F. MAb 17E10 binds at an approximately 56° tilt and 101F at a 32° tilt, in contrast to the other mAbs that are not cross-reactive and bind close to 0° tilt. The tilt angle may indeed be a determinant of cross-reactivity with hMPV F. Binding of 101F to pre-fusion F is dependent on the R429 residue and the G430A residues, while 17E10 bypasses R429 and utilizes G430 and I432. Therefore, the high tilt angle by 17E10 compared to 101F likely allows this residue switch interaction.

Site IV is a robust antigenic site for the RSV immune repertoire as evidenced by the activity of the potently neutralizing mAb 3M3, and it is one of two known antigenic sites on the RSV F protein that induces cross-reactive mAbs. Current thought in the RSV field is that the most potent neutralizing mAbs (D25, hRSV90) are directed toward the pre-fusion F conformation, particularly toward site Ø and site VIII. However, here we describe a very potent mAb 3M3 that is directed toward antigenic site IV, suggesting antigenic site IV may be useful for incorporation into next-generation vaccine formulations. A limitation of our study is the small number of mAbs generated. We cannot predict the frequency of 3M3-like or 17E10-like mAbs in the human repertoire, although a previous study has shown site IV mAbs in general represent a substantial portion of the human repertoire to RSV F [28]. These studies identify a new potent human mAb that could be used in prophylactic or therapeutic applications, and the data reveal important structural features of the F protein that could be used in rational structure-based vaccine design. For instance, as a vaccine antigen, the linear peptide that has been used as the canonical representation of antigenic site IV likely would be insufficiently immunogenic as a human vaccine antigen, however, incorporating residues slightly outside of this region and masking R429 may result in the production of potent and cross-neutralizing pan-Pneumovirus antibodies. Further structural analysis of cross-reactive site IV mAbs could facilitate development of a vaccine that protects against both RSV and hMPV.

## Materials and methods

### Ethics statement

Participation of healthy human adult subjects was approved by the Vanderbilt University Institutional Review Board, and blood samples were obtained only after informed written consent.

### Human hybridoma generation

Participation of healthy human adult subjects was approved by the Vanderbilt University Institutional Review Board, and blood samples were obtained only after informed consent. PBMCs were isolated from healthy human donor blood samples using Ficoll-Histopaque density gradient centrifugation. Approximately ten million PBMCs were mixed with 17 mL of transforming cell culture medium (Medium A, StemCell Technologies), 8 μg/mL of CpG (phosphorothioate-modified oligodeoxynucleotide ZOEZOEZZZZZOEEZOEZZZT, Invitrogen), 3 μg/mL of Chk2 inhibitor II (Sigma), 1 μg/mL of cyclosporine A (Sigma), and 4.5 mL of filtered supernatant from a culture of B95.8 cells (ATCC VR-1492) containing Epstein-Barr virus (EBV). After one week, transformed PBMCs were expanded into four 96-well culture plates using cell line expansion culture medium (Medium A containing 8 μg/mL CpG, 3 μg/mL Chk2 inhibitor II, and ten million irritated heterologous human PBMCs [Nashville Red Cross)]). After one week, culture supernatants were screened by ELISA against the post-fusion RSV A2 F protein. Cells from positive wells were fused with HMMA2.5 myeloma cells by electrofusion [30]. Fused cells were plated in 384-well plates in growth medium containing 100 μM hypoxanthine, 0.4 μM aminopterin, 16 μM thymidine (HAT Media Supplement, Sigma), and 7 μg/mL ouabain (Sigma). Hybridomas were screened after two weeks for mAb production by ELISA, and cells from wells with reactive supernatants were expanded to 96-well plates for one week before being screened again by ELISA, and then subjected to single-cell fluorescence-activated sorting. After cell sorting into 384-well plates containing Medium E (StemCell Technologies), hybridomas were screened by ELISA before expansion into both 48-well and 12-well plates.

### Enzyme-linked immunosorbent assay (ELISA) for binding to RSV F and hMPV F proteins

For recombinant protein capture ELISA, 384-well plates were treated with 2 μg/mL of antigen for one hour at 37°C or overnight at 4°C. Following this, plates were blocked for one hour with 2% milk supplemented with 2% goat serum. Primary mAbs and culture supernatants were applied to wells for one hour following three washes with PBS-T. Plates were washed with PBS-T four times before applying 25 μL secondary antibody (goat anti-human IgG Fc, Meridian Life Science) at a dilution of 1:4,000 in blocking solution. After a one-hour incubation, the plates were washed five times with PBS-T, and 25 μL of phosphatase substrate solution (1 mg/mL phosphatase substrate in 1 M Tris HCl pH 9.6, Sigma) was added to each well. The plates were incubated at room temperature before reading the optical density at 405 nm on a BioTek plate reader. ELISA experiments using biotintylated peptides were conducted by coating pre-blocked streptavidin-coated plates (Fisher) with 10 μg/mL peptide for two hours. After three washes with PBS-T, plates were coated with primary mAbs for one hour. The remaining steps were conducted as described above.

### Human mAb and Fab production and purification

Biologically cloned hybridoma cell lines were expanded in Medium E until approximately 80% confluent in 75-cm^2^ flasks. For antibody production, cells from one 75-cm^2^ cell culture flask were collected with a cell scraper and expanded to four 225-cm^2^ cell culture flasks in serum-free medium (Hybridoma-SFM, GIBCO). After 30 days, supernatants were sterile filtered using 0.45 μm pore size filter devices. For antibody purification, HiTrap MabSelectSure columns (GE Healthcare Life Sciences) were used to purify antibodies using the manufacturer’s protocol. To obtain Fab fragments, papain digestion was performed following the manufacturer’s protocol (Pierce Fab Preparation Kit, Thermo Scientific). Fab fragments were purified by removing IgG and Fc contaminants using a HiTrap MabSelectSure column (GE Healthcare Life Sciences).

### Production and purification or recombinant RSV F, hMPV F, motavizumab, mAb 101F, mAb 54G10, mAb MPE8, and mAb D25

Plasmids encoding cDNAs for pre-fusion (SC-TM) or post-fusion RSV subgroup A strain A2, and subgroup B strain 18537 pre-fusion (DsCav1, a gift from Barney Graham) were expanded in *E*. *coli* DH5α cells, and plasmids were purified using Qiagen Plasmid Maxiprep kits (Qiagen). Plasmids encoding cDNAs for the protein sequences of mAb 101F, mAb MPE8, and mAb D25, and motavizumab heavy and light chain sequences were cloned into vectors encoding human IgG1 and lambda or kappa light chain constant regions, respectively. Vectors encoding the heavy and light chain sequences of 54G10 were a gift from Dennis Burton. MAb 131-2a protein was obtained from Sigma. Commercial preparations of palivizumab (Medimmune) were obtained from the pharmacy at Vanderbilt University Medical Center. For each liter of protein expression, 1.3 mg of plasmid DNA was mixed with 2 mg of polyethylenimine in Opti-MEM I cell culture medium (Fisher). After 10 min, the DNA mixture was added to HEK293F cells (ThermoFisher R79007) at 1 x 10^6^ cells/mL. The culture supernatant was harvested after 5 days, and the protein was purified by HiTrap Excel column (GE Healthcare Life Sciences) for RSV F protein variants or HiTrap MabSelectSure columns for mAbs.

### Assays for competition-binding and peptide binding

All studies were conducted on an Octet Red FortéBio biolayer interferometry system using anti-penta-HIS biosensor tips for competition and streptavidin-coated sensors for peptide binding. For competition, an initial baseline was obtained in kinetics buffer (FortéBio, diluted 1:10 in PBS), followed by antigen loading with 20 μg/mL of his-tagged RSV F protein being immobilized onto biosensor tips for 120 s. The baseline signal was measured again for 60 s before biosensor tips were immersed into wells containing 100 μg/mL primary mAb for 300 s. Following this, biosensors were immersed into wells containing 100 μg/mL of a second mAb for 300 s. The percent binding of the second mAb in the presence of the first mAb was determined by comparing the maximum signal of the second mAb after the first mAb was added to the maximum signal of the second mAb alone. MAbs were considered non-competing if maximum binding of the second mAb was ≥ 66% of its un-competed binding. A level between 33%–66% of its un-competed binding was considered intermediate competition, and ≤ 33% was considered competing. To assess binding of mAbs to biotintylated peptides, streptavidin-coated sensors were immersed in kinetics buffer for 60 s, followed by immersion into biotintylated peptides at 5 μg/mL for 20 s. Following another 60 s baseline step, sensors were immersed in mAbs for 120 s for association. Dissociation from the peptide then was measured by immersing sensors in kinetics buffer for 120 s.

### Antibody epitope mapping

Shotgun mutagenesis epitope mapping of anti-RSV F antibodies was performed using an alanine scanning mutagenesis library for RSV F protein (RSV-A2; NCBI ref # FJ614814), covering 368 surface-exposed residues identified from crystal structures of both the pre-fusion and post-fusion conformations of RSV F. An RSV F expression construct was mutated to change each residue to an alanine (and alanine residues to serine). The resulting 368 mutant RSV F expression constructs were sequence confirmed and arrayed into a 384-well plate (one mutation per well). Library screening was performed as described previously [35]. The RSV F alanine scan library clones were transfected individually into human HEK-293T cells (ATCC CRL-3216) and allowed to express for 16 h before fixing cells in 4% (vol/vol) paraformaldehyde (Electron Microscopy Sciences) in PBS plus calcium and magnesium. Cells were incubated with mAbs, diluted in 10% (vol/vol) normal goat serum (NGS), for 1 h at room temperature, followed by a 30 min incubation with 3.75 μg/mL Alexa Fluor 488-conjugated secondary antibody (Jackson ImmunoResearch Laboratories) in 10% NGS. Cells were washed twice with PBS without calcium or magnesium and resuspended in Cellstripper (Cellgro) plus 0.1% BSA (Sigma-Aldrich). Cellular fluorescence was detected using a high-throughput flow cytometer (Intellicyt). Before library screening, to ensure that the signals were within the linear range of detection, the optimal screening concentrations for each mAb were determined using an independent immunofluorescence titration curve against cells expressing wild-type RSV F. Antibody reactivity against each mutant protein clone was calculated relative to wild-type protein reactivity by subtracting the signal from mock-transfected controls and normalizing to the signal from wild-type protein transfected controls. Mutations within clones were identified as critical to the mAb epitope if they did not support reactivity of the test mAb, but supported reactivity of other antibodies. This counter-screen strategy facilitates the exclusion of RSV F protein mutants that are misfolded or have an expression defect.

### RSV and hMPV neutralization experiments

Plaque reduction assays for RSV were conducted by first serially diluting mAbs, and incubating mAbs with a suspension of infectious RSV A2 or Long strain viruses for 1 hr. Following this, confluent HEp-2 (ATCC CCL-23) cell monolayers cultures in 24-well plates, maintained in Opti-MEM I (Fisher) supplemented with 2% fetal bovine serum at 37°C in a CO_2_ incubator, were inoculated with 50 μL of the antibody:virus mixture for 1 hr. After the hour, cells were overlaid with 1 mL of 0.75% methylcellulose dissolved in Opti-MEM I + 2% fetal bovine serum. Cells were incubated for four days, after which plaques were visualized by fixing cells with 10% neutral-buffered formalin and staining with crystal violet.

For the hMPV neutralization experiments, serially diluted mAbs were incubated for 1 hour with hMPV Jpn03-1 B2 strain. Vero cell (ATCC CCL-81) monolayers, maintained as described above, were overlaid with the mAb/virus mixture for one hour. Cells were overlaid with 0.75% methylcellulose dissolved in Opti-MEM + 0.0005% trypsin-EDTA. After five days, the cells were fixed with 10% neutral buffered formalin. Cells were immunostained by incubating with 1:1000 dilution each of anti-hMPV nucleoprotein antibody (Meridian Life Science C01851M) and anti-hMPV fusion protein antibody (Meridian Life Science C01852M) for one hour. After washing three times with water, the cells were incubated with 1:2000 of goat anti-mouse IgG (H+L) peroxidase labeled human serum absorbed antibody (SeraCare 074–1806) for one hour. After washing three times with water, the cells were overlaid with TrueBlue peroxidase substrate (SeraCare 54-78-00). Immunostained plaques were counted manually, and data from all neutralization experiments were analyzed with Prism software (GraphPad) to obtain IC_50_ values.

### Assembly and rescue of recombinant RSV A2-mKate2-R429A

Site-directed mutagenesis was performed on a subclone of the A2 F gene to introduce the R429A mutation using primers:

**429F**: 5′-GCATCCAATAAAAATGCTGGAATCATAAAGAC-3′ and
**429R**: 5′-GTCTTTATGATTCCAGCATTTTTATTGGATGC-3′

(Integrated DNA Technologies, Coralville, IA). Once sequence confirmed, the R429A A2 F gene was ligated into the bacterial artificial chromosome (BAC) pSynk-A2-mKate2 using SacII/SalI restriction sites. pSynk-A2-mKate2 contains the antigenomic cDNA of RSV A2 with an *mKate2* gene encoding the far-red monomeric Katushka-2 fluorescent reporter protein in the first position [36]. The recombinant virus was rescued by co-transfecting the RSV antigenomic BAC and four codon-optimized helper plasmids expressing the RSV L, N, M2-1 and P proteins into BSRT7/5 cells as previously described [37]. Master and working stocks were subsequently propagated and harvested in HEp-2 (ATCC CCL-23) cells [37–39], and the virus was titrated by immunodetection plaque assay as described [40].

### EM sample preparation and data collection

The complex was generated by mixing excess of human Fab with RSV F protein and incubation at 37°C for 1 h followed by size-exclusion chromatography (S200, 16/300; GE Healthcare Life Sciences) in 50 mM Tris pH 7.5, 50 mM NaCl. The sample (5 μl at 5 μg/ml) was applied to a glow-discharged copper grid coated with continuous carbon (EMS 400 mesh) for 1 min washed and blotted. Freshly prepared uranyl formate (0.75%) was added for 1min blotted and air dried as described [41].

Data collection was done using FEI Tecnai F20 microscope operated at 200kV and equipped with Gatan Ultrascan 4k x 4k CCD camera. The Random Comical Tilt (RCT) image pairs were acquired semi-automatically using SerialEM 3.6.3 [42] at nominal magnification of 50,000X with pixel size of 2.18A and defocus of 1.2–1.8 μm. The tilt pair was collected at -60° and 0°.

To generated 3D model of the RSV F-Fab complexes we used the RCT approach. Data processing was done using Scipion suit [43]. First tilt-pairs from the micrographs were picked using xmipp3—tilt pairs particle picking [44,45] with box size of 160 x 160 pixels. The particles were extract and binned by 2 to generate a box size of 80 x 80 pixels (4.36 A/px). The images were normalized using xmipp3—extract particle pairs [44,45]. The untilted particles were band-pass filtered before aligning and classification using xmipp3 –cl2d [44,45]. From the 2D analysis we selected well-aligned classes with clear RSV-F protein-Fab complexes and performed 3D reconstruction using the corresponding tilted particles using xmipp3—random conical tilt [44,45]. This initial model was then used for further 3D refinement using both the tilted images and 10% of the untilted particles in RELION—3D auto-refine [46]. Volume display and figures were done in UCSF Chimera [47].

## Supporting information

S1 Table Summary of binding characteristics for the studies mAbs. The summary of neutralization and binding data is provided for reference. A green color and “Yes” indicates the mAb does neutralize or bind to each protein indicated. A red color and “No” indicates no binding or neutralization. An orange color and “partial” indicates some loss of binding. The binding angle relative to antigenic site IV is indicated. No data is available for those boxes in grey with n.d. (not determined).
(PDF)

S1 Fig Summary of known antigenic sites on the RSV F protein. (PDF)

S2 Fig ELISA binding curves for the newly generated site IV mAbs and controls to RSV F protein and construct variants. EC_50_ values for these curves are displayed in Table 1. Zika NS1 protein was used as a negative control. Each data point is the average of three independent experiments, each with four technical replicates. Error bars represent the standard deviation.
(PDF)

S3 Fig Neutralization curves for the newly generated site IV mAbs and controls. IC_50_ values are displayed in Table 1. An Ebola virus-specific mAb EBOV284 was included as a negative control. Data points indicate the average of three technical replicates. Error bars represent the standard deviation.
(PDF)

S4 Fig Critical residues for mAb 17E10 binding. (**A**) A shotgun mutagenesis mutation library for RSV F protein encompassing 368 mutations, where each amino acid was individually mutated to alanine, was constructed. Each well contained one mutant with a defined substitution. Reactivity results for a representative 384-well plate are shown. Eight positive (wild-type RSV F) and eight negative (mock-transfected) control wells were included on each plate. (**B**) Human HEK-293T cells cells expressing the RSV F mutation library were tested for immunoreactivity with 17E10, which was measured using an Intellicyt high-throughput flow cytometer. Using algorithms described elsewhere [35], clones with reactivity of <30% relative to that of wild-type RSV F yet >70% reactivity for a different RSV F mAb were identified to be critical for 17E10 binding. (**C**) Critical residues identified for 3M3, 6F18, and 17E10 are listed with the mean binding reactivies for each mAb as well as control antibodies palivizumab and D25. Reactivities are expressed as a percentage of the reactivity of the wild type with ranges (maximum minus minimum values) given in parentheses. Values shaded in gray are for critical residues. Data shown is the average of two replicate values.
(PDF)

S5 Fig ELISA binding curves for the site IV mAbs and controls to (A) pre-fusion (SC-TM) or (B) post-fusion RSV A2 mutant proteins. Each data point is the average of three independent experiments, each with four technical replicates. Error bars indicate the standard deviation. EC_50_ values are shown in Fig 2C.
(PDF)

S6 Fig Plaque-reduction assay curves for the newly generated site IV mAbs and controls for neutralization of the RSV A2 R429A mutant virus. IC_50_ values are displayed in Fig 2C. Data points indicate the average of three technical replicates. Error bars represent the standard deviation.
(PDF)

S7 Fig ELISA binding curves of site IV mAbs to biotinylated site IV 15-mer peptides coated on streptavidin ELISA plates. Data points are the average of two technical replicates. Error bars indicate the range of the two measurements.
(PDF)
