# Supplementary material for: Human antibody recognition of antigenic site IV on Pneumovirus fusion proteins
Source: PLoS Pathog. 2018 Feb 22;14(2):e1006837. doi: 10.1371/journal.ppat.1006837 (PMC5823459; doi:10.1371/journal.ppat.1006837)
Supplement: S1 Table — The summary of neutralization and binding data is provided for reference. A green color and “Yes” indicates the mAb does neutralize or bind to each protein indicated. A red color and “No” indicates no binding or neutralization. An orange color and “partial” indicates some loss of binding. The binding angle relative to antigenic site IV is indicated. No data is available for those boxes in grey with n.d. (not determined). (PDF) [file ppat.1006837.s001.pdf]

Table S1. Summary of binding characteristics for the mAbs studied.

mAb	Cross-Reactive?	Binding Angle	R429A Neut	Cell-surface			prefusion		postfusion			peptide					
				R429	G430	I432	R429	G430	R429	G430	I432	15-mer WT	15-mer R429	15-mer G430	15-mer I432	30-mer A	30-mer B
2N6	No	-6	Yes	Yes	Yes	Yes	Yes	Yes	Yes	Yes	Yes	No	No	No	No	Yes	No
3M3	No	6	No	No	Yes	Yes	No	Yes	No	Yes	Yes	No	No	No	No	No	No
6F18	No	-6	No	No	Yes	Yes	No	Yes	Yes	Yes	Yes	No	No	No	No	No	No
17E10	Yes	-56	Yes	Yes	No	No	Yes	partial	Yes	Yes	Yes	Yes	No	No	No	Yes	Yes
101F	Yes	-32	No	n.d.	n.d.	n.d.	No	partial	Yes	Yes	Yes	Yes	No	No	No	Yes	Yes
54G10	Yes	n.d.	No	n.d.	n.d.	n.d.	No	No	Yes	No	Yes	No	No	No	No	No	No

The summary of neutralization and binding data is provided for reference. A green color and "Yes" indicates the mAb does neutralize or bind to each protein indicated. A red color and "No" indicates no binding or neutralization. An orange color and "partial" indicates some loss of binding. The binding angle relative to antigenic site IV is indicated. No data is available for those boxes in grey with n.d. (not determined).
