# Supplementary figures and images for: Human antibody recognition of antigenic site IV on Pneumovirus fusion proteins
Source: PLoS Pathog. 2018 Feb 22;14(2):e1006837. doi: 10.1371/journal.ppat.1006837 (PMC5823459; doi:10.1371/journal.ppat.1006837)

## Supporting Information.

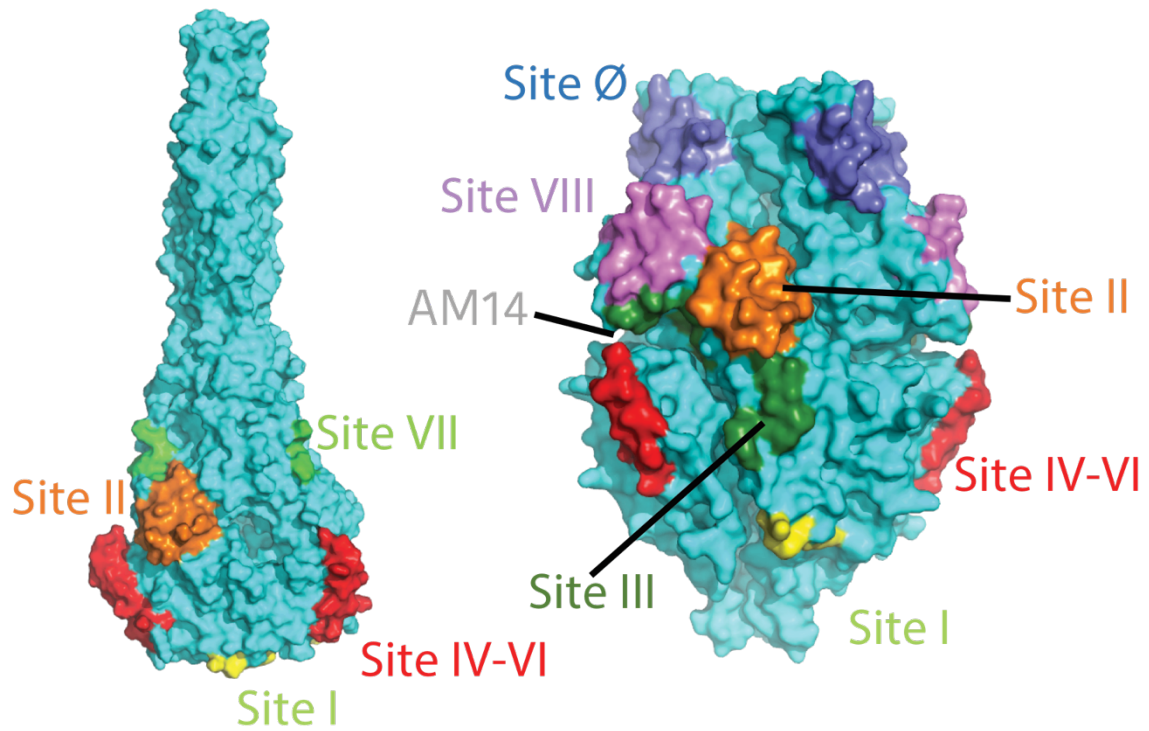

**Fig. S1. Summary of known antigenic sites on the RSV F protein.**

Supplement: S1 Fig — (PDF) [file ppat.1006837.s002.pdf]
