# Supplementary material for: Human antibody recognition of antigenic site IV on Pneumovirus fusion proteins
Source: PLoS Pathog. 2018 Feb 22;14(2):e1006837. doi: 10.1371/journal.ppat.1006837 (PMC5823459; doi:10.1371/journal.ppat.1006837)
Supplement: S2 Fig — EC50 values for these curves are displayed in Table 1. Zika NS1 protein was used as a negative control. Each data point is the average of three independent experiments, each with four technical replicates. Error bars represent the standard deviation. (PDF) [file ppat.1006837.s003.pdf]

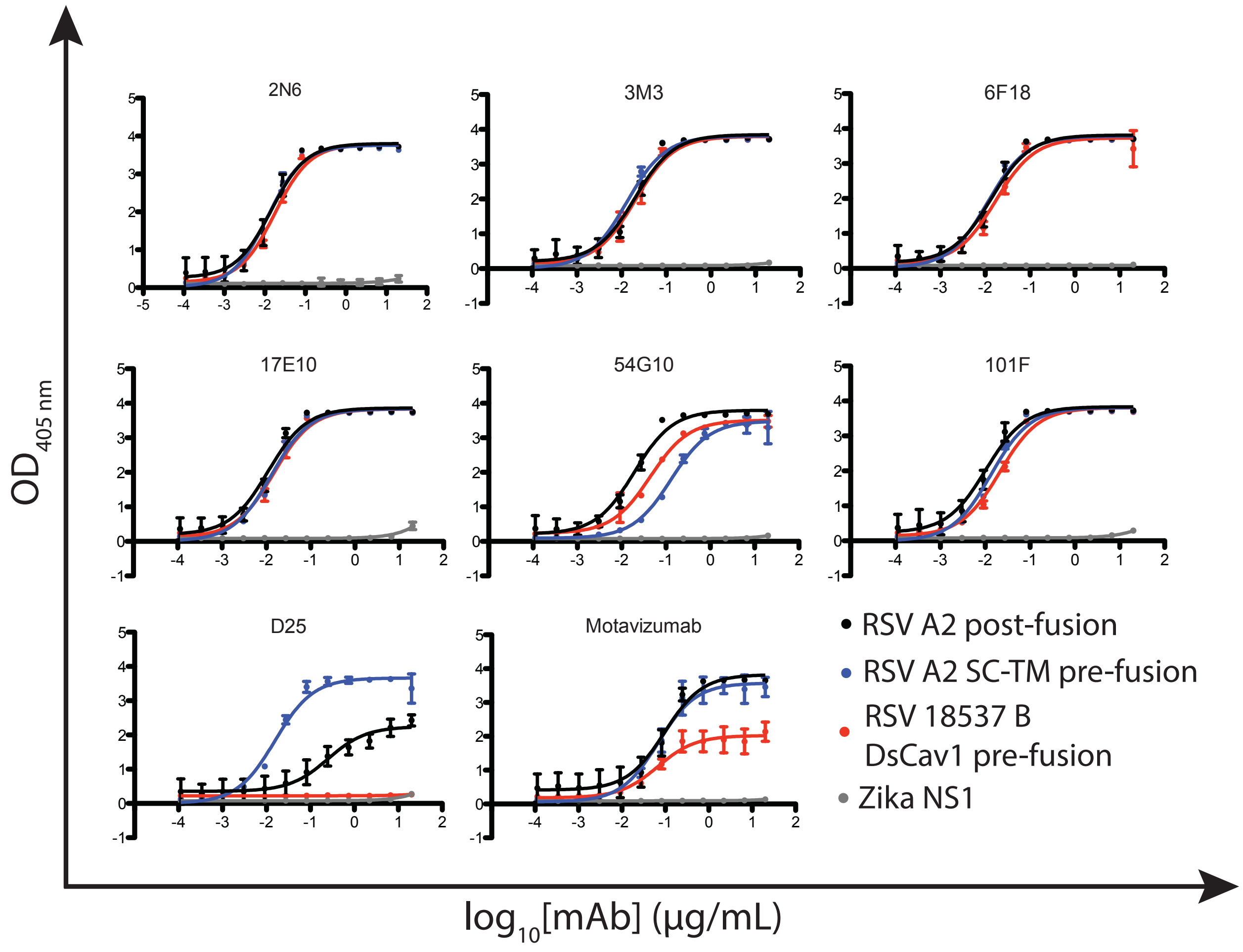

Fig. S2. ELISA binding curves for the newly generated site IV mAbs and controls to RSV F protein and construct variants. EC_{50} values for these curves are displayed in Table 1. Zika NS1 protein was used as a negative control. Each data point is the average of three independent experiments, each with four technical replicates. Error bars represent the standard deviation.
