# Supplementary material for: Human antibody recognition of antigenic site IV on Pneumovirus fusion proteins
Source: PLoS Pathog. 2018 Feb 22;14(2):e1006837. doi: 10.1371/journal.ppat.1006837 (PMC5823459; doi:10.1371/journal.ppat.1006837)
Supplement: S3 Fig — IC50 values are displayed in Table 1. An Ebola virus-specific mAb EBOV284 was included as a negative control. Data points indicate the average of three technical replicates. Error bars represent the standard deviation. (PDF) [file ppat.1006837.s004.pdf]

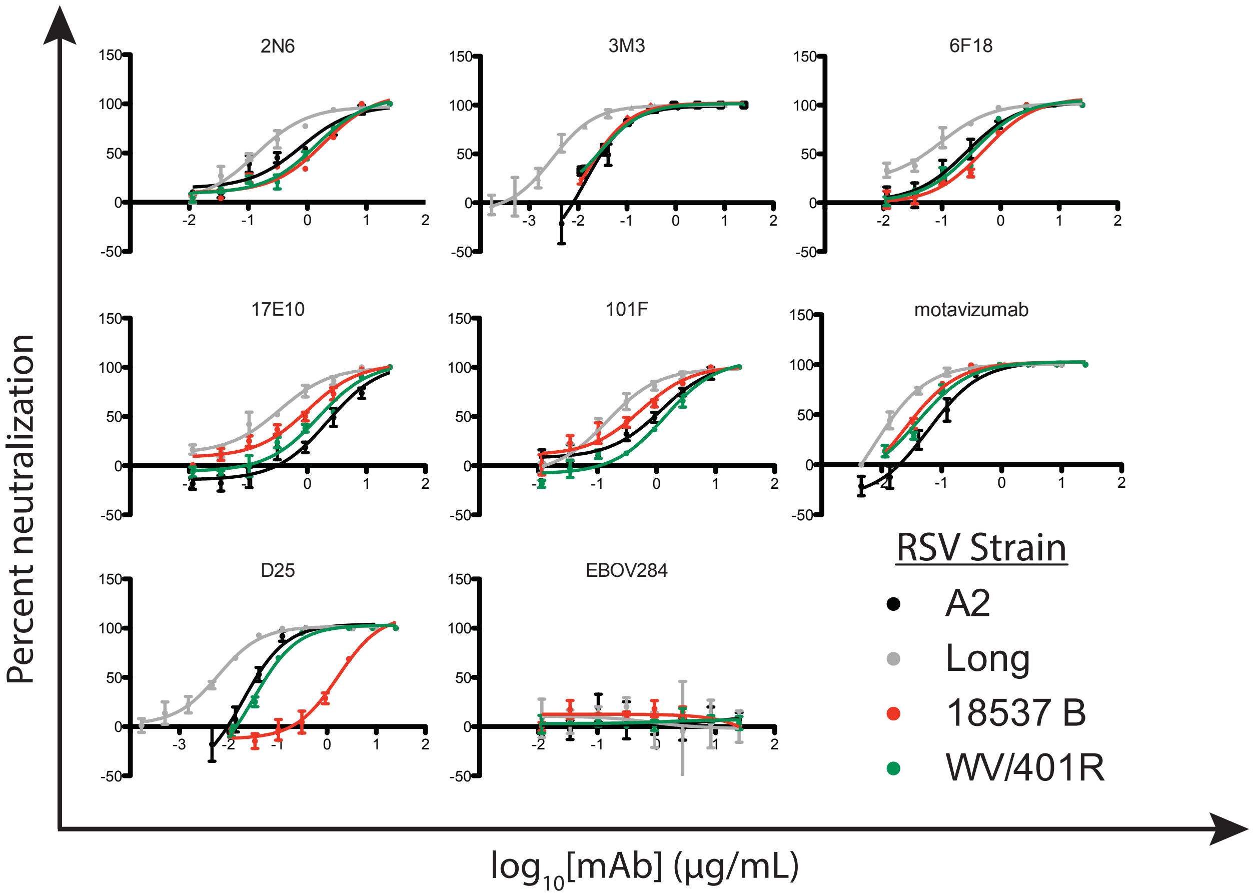

Fig S3. Neutralization curves for the newly generated site IV mAbs and controls. IC_{50} values are displayed in Table 1. An Ebola virus-specific mAb EBOV284 was included as a negative control. Data points indicate the average of three technical replicates. Error bars represent the standard deviation.
