# Supplementary material for: Human antibody recognition of antigenic site IV on Pneumovirus fusion proteins
Source: PLoS Pathog. 2018 Feb 22;14(2):e1006837. doi: 10.1371/journal.ppat.1006837 (PMC5823459; doi:10.1371/journal.ppat.1006837)
Supplement: S4 Fig — (A) A shotgun mutagenesis mutation library for RSV F protein encompassing 368 mutations, where each amino acid was individually mutated to alanine, was constructed. Each well contained one mutant with a defined substitution. Reactivity results for a representative 384-well plate are shown. Eight positive (wild-type RSV F) and eight negative (mock-transfected) control wells were included on each plate. (B) Human HEK-293T cells cells expressing the RSV F mutation library were tested for immunoreactivity with 17E10, which was measured using an Intellicyt high-throughput flow cytometer. Using algorithms described elsewhere [35], clones with reactivity of <30% relative to that of wild-type RSV F yet >70% reactivity for a different RSV F mAb were identified to be critical for 17E10 binding. (C) Critical residues identified for 3M3, 6F18, and 17E10 are listed with the mean binding reactivies for each mAb as well as control antibodies palivizumab and D25. Reactivities are expressed as a percentage of the reactivity of the wild type with ranges (maximum minus minimum values) given in parentheses. Values shaded in gray are for critical residues. Data shown is the average of two replicate values. (PDF) [file ppat.1006837.s005.pdf]

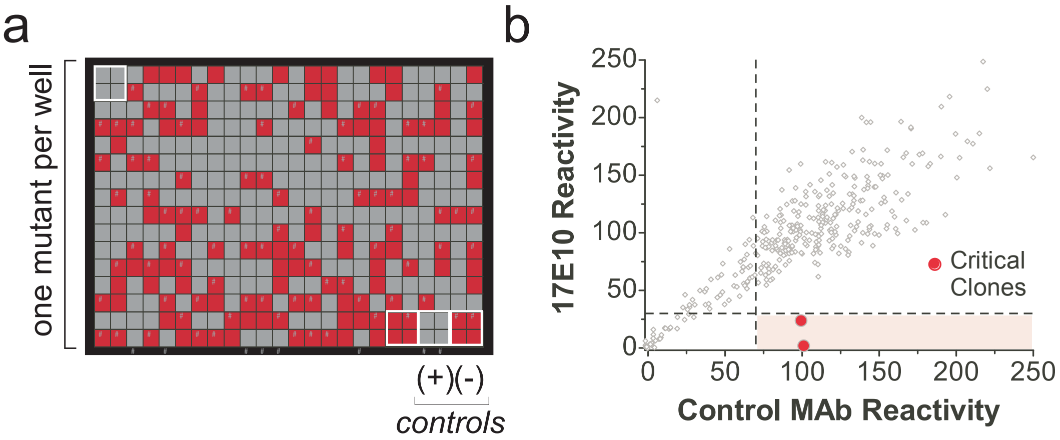

c

	Binding Reactivity (% WT)					
Mutation	2N6	3M3	6F18	17E10	palivizumab	D25
R429A	209.2 (14)	6.1 (7)	1.6 (0)	214.9 (33)	311.1 (86)	385.0 (13)
G430A	207.4 (81)	101.1 (55)	137.1 (59)	2.0 (0)	397.4 (31)	308.1 (224)
I432A	125.6 (4)	99.4 (69)	57.6 (20)	23.9 (14)	261.7 (51)	237.8 (161)

Fig S4. Critical residues for mAb 17E10 binding. (a) A shotgun mutagenesis mutation library for RSV F protein encompassing 368 mutations, where each amino acid was individually mutated to alanine, was constructed. Each well contained one mutant with a defined substitution. Reactivity results for a representative 384-well plate are shown. Eight positive (wild-type RSV F) and eight negative (mock-transfected) control wells were included on each plate. (b) Human HEK-293T cells expressing the RSV F mutation library were tested for immunoreactivity with 17E10, which was measured using an Intellicyt high-throughput flow cytometer. Using algorithms described elsewhere [35], clones with reactivity of <30% relative to that of wild-type RSV F yet >70% reactivity for a different RSV F mAb were identified to be critical for 17E10 binding. (c) Critical residues identified for 3M3, 6F18, and 17E10 are listed with the mean binding reactivities for each mAb as well as control antibodies palivizumab and D25. Reactivities are expressed as a percentage of the reactivity of the wild type with ranges (maximum minus minimum values) given in parentheses. Values shaded in gray are for critical residues. Data shown is the average of two replicates values.
