# Supplementary material for: Human antibody recognition of antigenic site IV on Pneumovirus fusion proteins
Source: PLoS Pathog. 2018 Feb 22;14(2):e1006837. doi: 10.1371/journal.ppat.1006837 (PMC5823459; doi:10.1371/journal.ppat.1006837)
Supplement: S5 Fig — Each data point is the average of three independent experiments, each with four technical replicates. Error bars indicate the standard deviation. EC50 values are shown in Fig 2C. (PDF) [file ppat.1006837.s006.pdf]

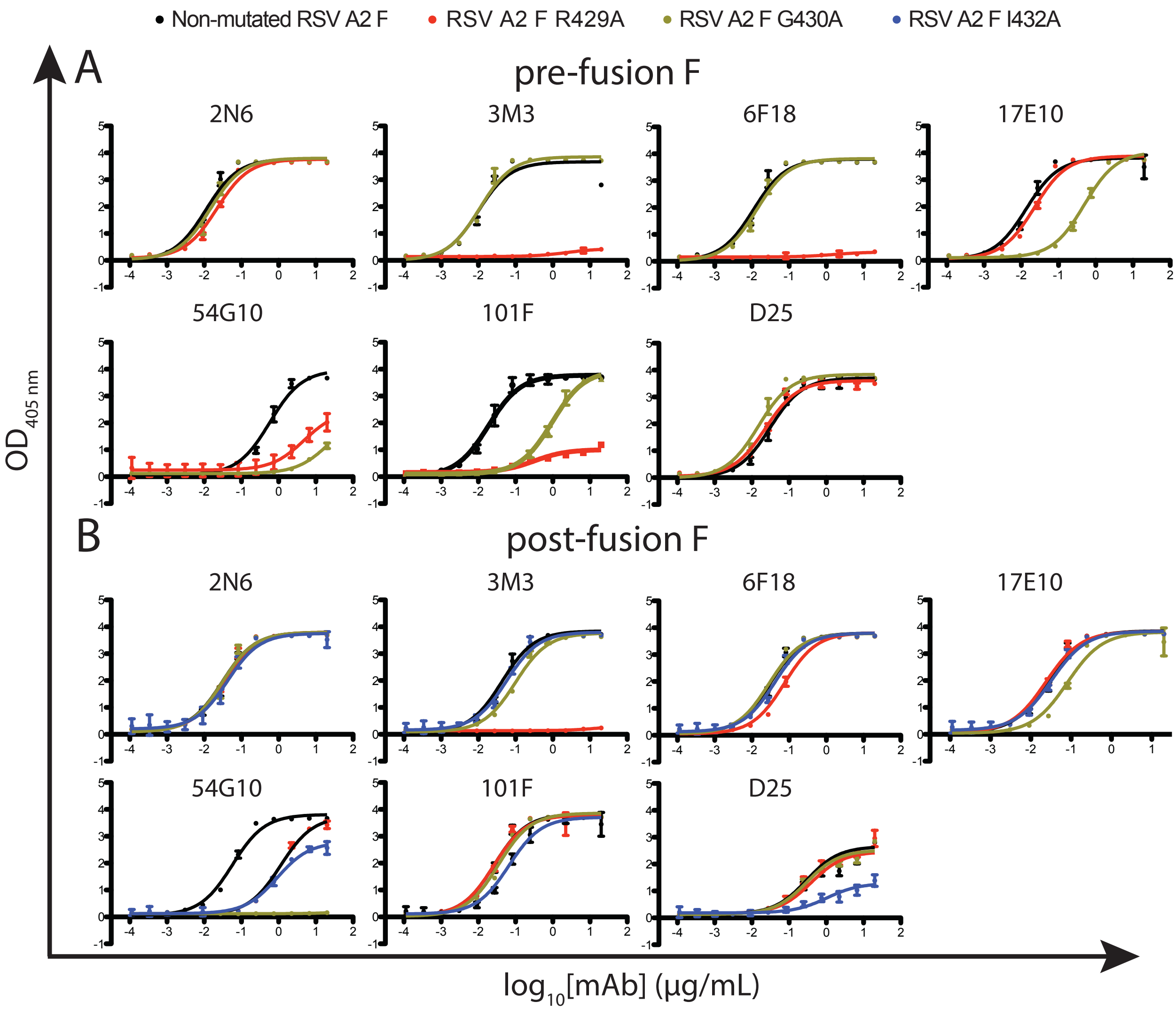

Fig. S5. ELISA binding curves for the site IV mAbs and controls to (A) pre-fusion (SC-TM) or (B) post-fusion RSV A2 mutant proteins. Each data point is the average of three independent experiments, each with four technical replicates. Error bars indicate the standard deviation. EC_{50} values are shown in Figure 2C.
