# Supplementary material for: Human antibody recognition of antigenic site IV on Pneumovirus fusion proteins
Source: PLoS Pathog. 2018 Feb 22;14(2):e1006837. doi: 10.1371/journal.ppat.1006837 (PMC5823459; doi:10.1371/journal.ppat.1006837)
Supplement: S7 Fig — Data points are the average of two technical replicates. Error bars indicate the range of the two measurements. (PDF) [file ppat.1006837.s008.pdf]

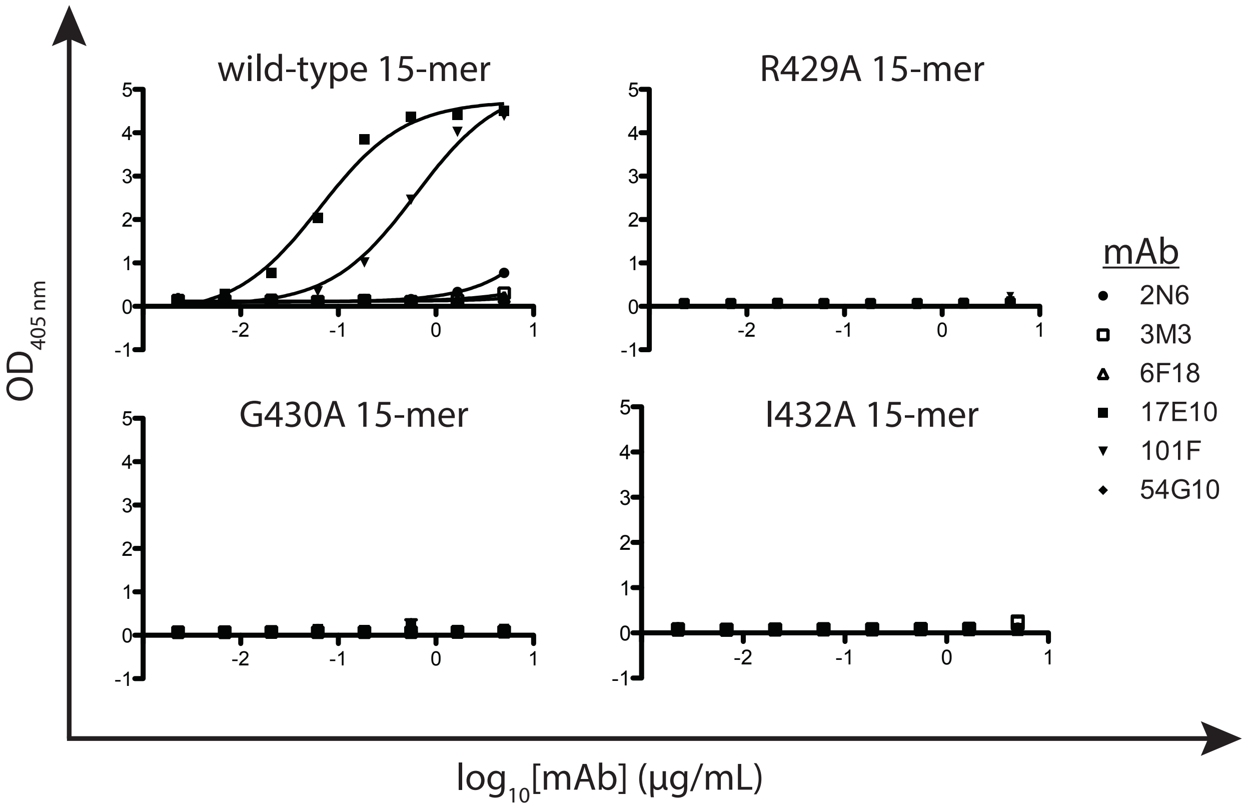

Fig. S7. ELISA binding curves of site IV mAbs to biotinylated site IV 15-mer peptides coated on streptavidin ELISA plates. Data points are the average of two technical replicates. Error bars indicate the range of the two measurements.
